# mGem: How many fungal secondary metabolites are produced by filamentous fungi? Conservatively, at least 1.4 million

**DOI:** 10.1128/mbio.01381-25

**Published:** 2025-09-16

**Authors:** Olivia L. Riedling, Antonis Rokas

**Affiliations:** 1 Department of Biological Sciences, Vanderbilt University 5718, Nashville, Tennessee, USA; 2 Evolutionary Studies Initiative, Vanderbilt University 5718, Nashville, Tennessee, USA; Instituto Carlos Chagas, Curitiba, Brazil

**Keywords:** *Aspergillus*, fungi, secondary metabolites, specialized metabolites, diversity, biosynthetic gene clusters, natural products, pezizomycotina

## Abstract

The ~30,000 known fungal secondary metabolites (SMs) are a vital component of the bioeconomy. SMs are biosynthesized by biosynthetic gene clusters (BGCs), i.e., sets of genes in close physical proximity in the genome. The bulk of these SMs are produced by filamentous fungi in the Pezizomycotina subphylum (phylum Ascomycota). To gauge the magnitude of chemodiversity in this subphylum, we utilized data from the well-characterized genus *Aspergillus* and a previous Pezizomycotina genomic survey. With 30–50 BGCs per genome, our rarefaction analyses show that the ~85,000 known species in Pezizomycotina likely contain 1.4–4.3 million SMs from an estimated 870 thousand to 2.7 million gene cluster families. Considering that only 5% of fungal species have been described and that the actual number of Pezizomycotina species is likely closer to a million, the projected number of SMs is likely between ~16 and 50 million. These estimates suggest that most fungal SMs remain undiscovered.

## PERSPECTIVE

**Fig 1:**
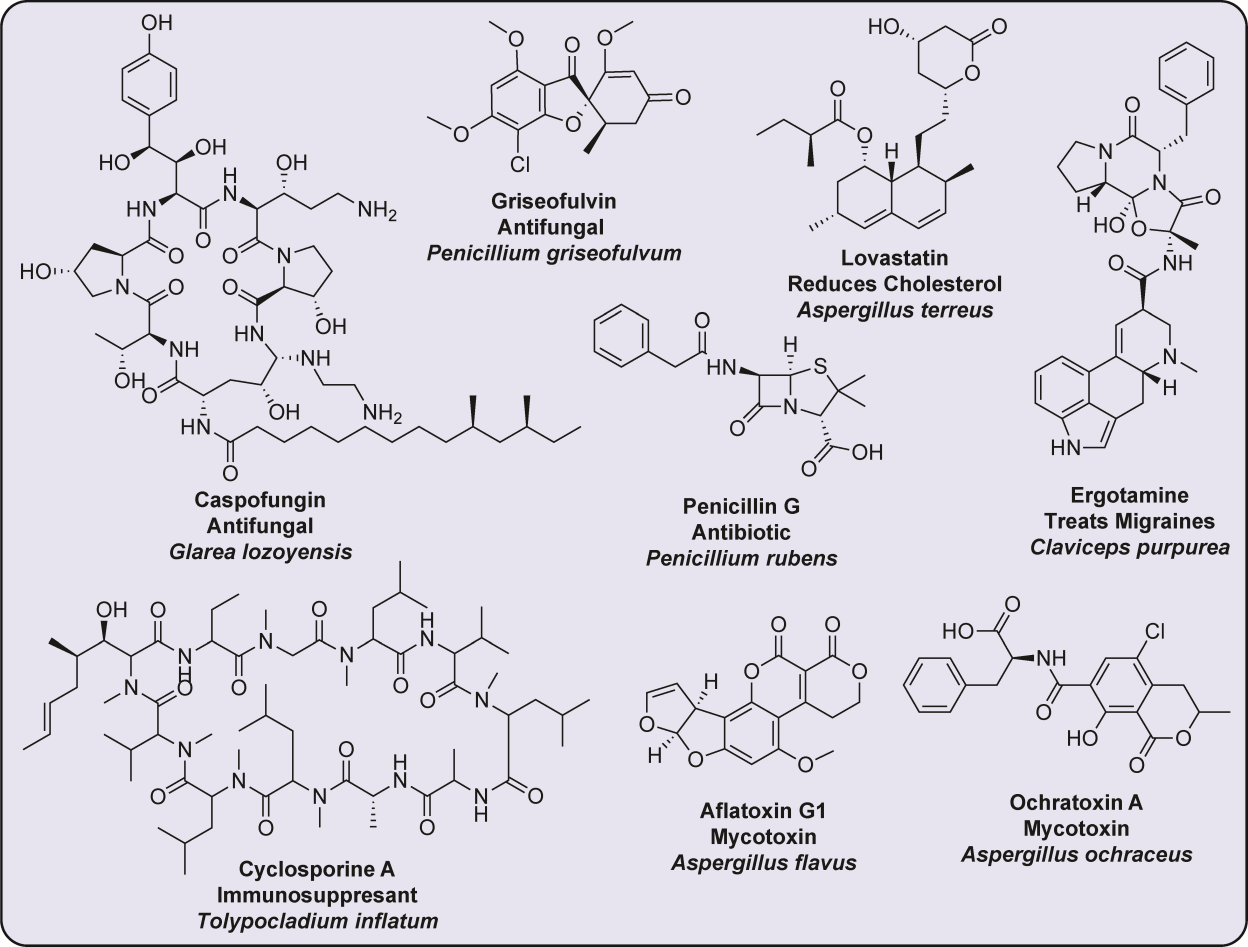
SMs produced by filamentous fungi that are relevant to the bioeconomy. Fungal SMs play a crucial economic role, being harnessed in diverse industries while also posing potential health risks. In the pharmaceutical sector, they have been instrumental in developing life-saving drugs: for example, lovastatin produced by *Aspergillus terreus* is used as a cholesterol-lowering agent (11); antifungals like griseofulvin and caspofungin produced by *Penicillium griseofulvum* (12*)* and *Glarea lozoyensis* (13)*,* respectively, are used to treat fungal infections; and the iconic antibiotic penicillin G isolated from *P. rubens* revolutionized bacterial infection management (2). Ergotamine, produced by *Claviceps purpurea*, is used to treat migraines and postpartum hemorrhages (14), while the immunosuppressant cyclosporine A produced by *Tolypocladium inflatum* is utilized in organ transplantation (15). While many fungal SMs are beneficial, the production of many others poses substantial human and animal health risks: for example, the potent carcinogens aflatoxin B1 from *A. flavus* (16) and ochratoxin A from *A. ochraceus* (17) are both commonly found in fungal-contaminated food products.

Since the discovery of penicillin in 1928, fungi have been at the forefront of natural product discovery (1, 2). To date, over 30,000 fungal secondary metabolites (SMs), small biologically active compounds, have been characterized (3). This set of known fungal SMs includes the biomedically important SMs lovastatin (4), a cholesterol-lowering drug; cyclosporine (5), and mycophenolic acid (6), which possess immunosuppressant properties; and penicillin (1), the world’s first antibiotic (Fig. 1). Fungal SMs also have a strong presence in the bioeconomy for natural food colorants (e.g., *Monascus* pigments like monascorubrine), cosmetics (lentinan, from *Lentinula edodes,* protects skin against environmental pollutants), and biocontrol agents (the strobilurin fungicides) (7). In addition to the biomedically relevant SMs, fungi are notorious producers of mycotoxins, which can result in mold toxicity, autoimmune diseases, and cancer (8–10), necessitating systematic efforts to monitor fungal plant diseases and fungal crop/grain contamination (Fig. 1).

The fungal lineage responsible for most of the known SMs is the Pezizomycotina subphylum in the phylum Ascomycota. The subphylum contains about 85,000 described species, including species from well-studied genera that are iconic producers of SMs, such as *Fusarium*, *Alternaria*, *Penicillium*, *Aspergillus*, and *Cordyceps* (18). The genes necessary for the biosynthesis of SMs are typically organized into biosynthetic gene clusters (BGCs) (19). BGCs are highly diverse in their gene content and organization and typically exhibit narrow taxonomic distributions (e.g., species- and genus-specific distributions of SMs are well known). The high interspecific diversity of BGCs, coupled with the ability of a single BGC to biosynthesize numerous SMs, suggests that the magnitude of fungal SM diversity (or chemodiversity) may be very large (20, 21).

To estimate the actual number of SMs produced by fungi across the Pezizomycotina subphylum, we utilized rarefaction analyses using data from the well-characterized fungal genus *Aspergillus* and from a previous genomic survey of Pezizomycotina (20) as guides. Our projections suggest there are at least 1.4 million SMs in the ~85,000 known species in the Pezizomycotina subphylum, which means that for every known SM (~30,000), there are potentially ~50–100 additional SMs that have yet to be unearthed. These estimates of SMs are likely on the low end, considering that only an estimated 3–8% of fungal species (including Pezizomycotina species) have been described and that numerous BGCs can produce multiple SMs. We conclude that the vast majority of fungal chemodiversity awaits discovery.

## *ASPERGILLUS* LIKELY HARBORS ~18,000 SMs, A NUMBER ON PAR WITH THAT OF KNOWN FUNGAL SMs

The genus *Aspergillus* has ~450 described species and has been very well characterized with respect to its secondary metabolism (22–26), genomics (27–30), and taxonomy (31–33). There are more than 100 *Aspergillus* species with publicly available genomes, and around 150 *Aspergillus* BGCs have been experimentally linked to their respective SMs (24). The availability of high-quality genomic, natural product, and taxonomic data makes *Aspergillus* an excellent model for estimating both the number of SMs produced by a typical filamentous fungal species and the number of BGCs present in its genome.

**Fig 2:**
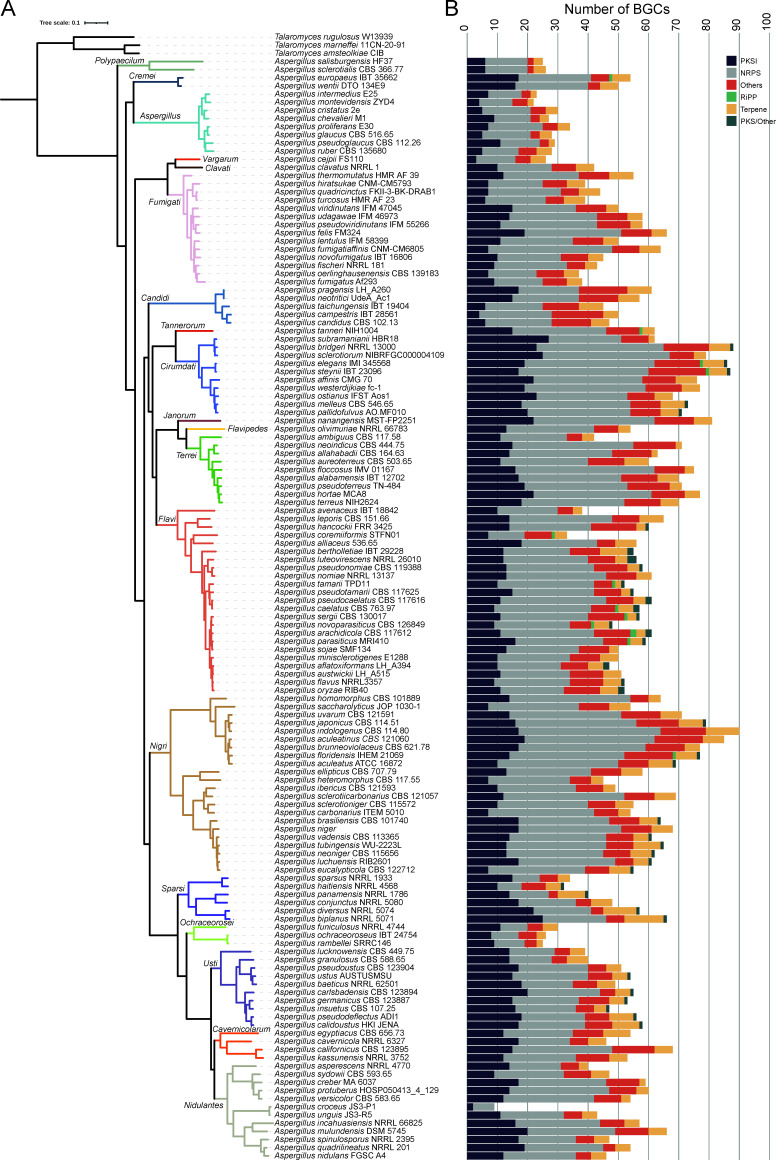
*Aspergillus* species display diverse BGCs and classes of SMs. (**A**) Phylogenetic tree of genomic data from 135 *Aspergillus* species used in this study, which represent nearly a third of *Aspergillus* species diversity. The clade colors and labels correspond to the taxonomic section within the genus. (**B**) Putative number of BGCs per genome and their predicted classes. The *X*-axis displays the number of BGCs, and the *Y*-axis displays the species. The color corresponds to the putative class of the BGC.

We used the antiSMASH (34) software to predict BGCs across 135 *Aspergillus* genomes and subsequently used the BiG-SCAPE (35) software to group putative BGCs into gene cluster families (GCFs). We identified a total of 6,972 putative BGCs after filtering (an average of 51.6 per *Aspergillus* species), which grouped into 4,463 GCFs. Conservation of BGCs across species was low; 3,607/4,463 (or ~80%) of GCFs were present in only one species, and just 34 families were present in 10 or more species. We further found that *Aspergillus* species generally produce diverse classes of SMs (Fig. 2).

**Fig 3:**
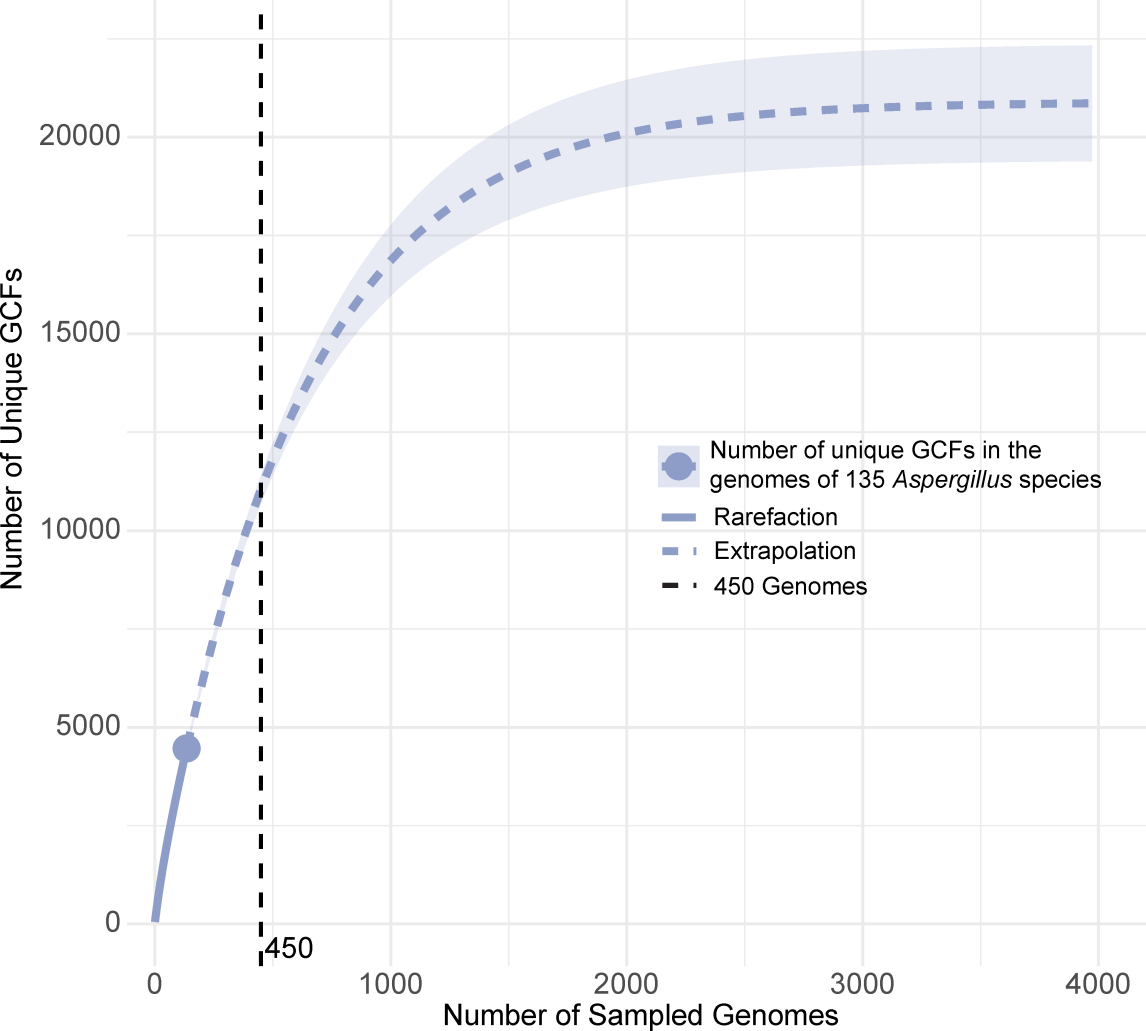
The genomes of species in the genus *Aspergillus* are estimated to contain a great richness of BGCs. The X-axis displays the number of sampled *Aspergillus* genomes, and the *Y*-axis shows the number of unique GCFs. The solid purple line in the rarefaction curve based on the genomes of 135 *Aspergillus* species, the purple circle corresponds to the number of unique GCFs (4,463) in the sampled genomes of *Aspergillus* species (135), and the dashed line is the extrapolation of the number of unique GCFs as the number of sampled genomes increases. Shaded purple regions denote the 95% confidence intervals. The dashed black line indicates the estimated number of unique GCFs (~11,500) for the number of currently described *Aspergillus* species (450).

To estimate the actual number of SMs likely to be produced by *Aspergillus* fungi*,* we used the BiG-SCAPE data and generated a rarefaction curve (36) based on the presence and absence of each GCF across the 135 *Aspergillus* species. Aligning our estimates with the current number of described *Aspergillus* species (450) suggests that the genus is estimated to contain ~11,500 GCFs (Fig. 3).

The Minimum Information about a Biosynthetic Gene Cluster (MIBiG) database (24) contains 158 *Aspergillus* BGCs paired with their corresponding SM(s); across these 158 BGCs, we calculated the effective value of SMs per BGC by down-weighting shared SMs. We found the average effective number of SMs per BGC in *Aspergillus* was about 1.57. Using this value, we estimate that the ~11,500 GCFs likely present in the 450 known species of *Aspergillus* can potentially produce ~18,055 SMs. Given that many BGCs can yield numerous SMs like the aflatoxins (37), chaetoglobosins (38), fumiquinazolines (39), and azanigerones (40), the estimate of 1.57 SMs per BGC likely falls short of the true number of SMs produced by Pezizomycotina.

Currently, there are 4,247 *Aspergillus* SMs in the Dictionary of Natural Products (41) and 3,100 SMs in the Natural Products Atlas (42). Our estimates of ~18,000 SMs imply that we have only characterized 10%–20% of *Aspergillus* SMs, suggesting that the known species in this genus alone harbor a large reservoir of undiscovered SMs.

## MILLIONS OF FUNGAL SECONDARY METABOLITES AWAIT DISCOVERY

Building on our estimates of the diversity of BGCs and SMs in *Aspergillus,* we next projected the number of SMs across the Pezizomycotina subphylum. As the number of BGCs varies across genera and is not as well characterized as in *Aspergillus*, we used three BGC/species estimates: (i) a conservative estimate of 30 BGCs per species, (ii) a previously published estimate of 40 BGCs per species based on an analysis of 721 genomes from ~480 Pezizomycotina species (20), and (iii) an estimate of 50 BGCs per species based on our *Aspergillus* data; extrapolating to the 85,000 known species of Pezizomycotina, we infer that filamentous fungi contain between 2,550,000 and 4,250,000 BGCs.

To refine these projections, we next sought to estimate the proportion of BGCs that are the first occurrence of their respective GCF from the total number of Pezizomycotina BGCs (i.e., the GCF/BGC discovery ratio). In *Aspergillus*, the 6,972 total predicted BGCs were grouped into 4,463 GCFs, suggesting that there are roughly 1.5 times more clusters than cluster families (or, put differently, roughly two-thirds or 64% of BGCs are the first instance of their GCF). To supplement the estimate of GCFs based on *Aspergillus*, we also made estimates based on the 721 Pezizomycotina genomes used by Robey et al. (20). That study found on average 39.8 BGCs per genome and 9,808 GCFs in Pezizomycotina (i.e., approximately 28,696 BGCs), suggesting that there are three times more clusters (34.2%) than families across the genomes (Table 1).

**TABLE 1:** Data-rooted estimates of GCFs and SMs based on 135 *Aspergillus* genomes and 721 Pezizomycotina genomes

GCF/BGC discovery ratios	No. of GCFs			No. of SMs			Reference
	Low	Avg	High	Low	Avg	High	
64% (based on 135 *Aspergillus* genomes)	1,632,336	2,176,449	2,720,561	2,562,768	3,417,024	4,271,280	This study
34% (based on 721 Pezizomycotina genomes)	871,564	1,162,085	1,452,607	1,368,355	1,824,474	2,280,592	(20)

Applying the Pezizomycotina and *Aspergillus* GCF/BGC discovery ratios, we generated scaled estimates of GCFs from the average number of BGCs/species and then multiplied these values by the known number of Pezizomycotina species and by 1.57 SMs per BGC. From these calculations, we project that the ~85,000 species of Pezizomycotina harbor between ~871,564 and ~2,720,561 GCFs responsible for the biosynthesis of a potential ~1.4 million to ~4.3 million SMs (Table 1). These numbers highlight the immense and largely unexplored chemodiversity of even the known species of filamentous fungi, underscoring the vast potential for discovery.

## THE UNTAPPED POTENTIAL OF SECONDARY METABOLITES ACROSS FILAMENTOUS FUNGI

Current estimates suggest we have characterized only about 3–8% of all fungal species (43). The Catalogue of Life lists ~156,000 fungal species, of which approximately 85,000 are within Pezizomycotina (44), which corresponds to ~54% of known species. Given that total fungal diversity is estimated to be between 2.2 and 3.8 million species (43), the true number of species in this subphylum likely exceeds the million species mark, in which case the projected number of fungal SMs could very well be in the dozens of millions range (based on our most conservative estimates, i.e., assuming 30 BGCs/species and a GCF/BGC discovery ratio of 34%, one million species may likely harbor more than 16 million SMs).

Finally, to gain insights into the chemical diversity of SMs, we analyzed the diversity of the chemical structures of SMs linked to all fungal BGCs in the MIBiG database (i.e., BGC-SM pairs from *Aspergillus* and approximately 100 other fungal genera). We calculated Tanimoto similarity based on the molecular fingerprints of each SM and a diversity (*d*) metric (1 – mean similarity across all compounds). Values of *d* near 0 indicate high similarity in chemical structures, whereas values of *d* near 1 indicate a high degree of diversity. We found that *d* = 0.88 for *Aspergillus* (*n *= 221) and *d* = 0.89 for Pezizomycotina (including *Aspergillus*, *n *= 785), respectively, suggesting that the millions of SMs across Pezizomycotina are likely to be highly diverse.

To contextualize our structural diversity estimates (*d* = 0.88 for *Aspergillus* and *d* = 0.89 for Pezizomycotina fungi), we calculated comparable diversity values for bacterial SMs curated in the MIBiG database. We found that bacterial SMs exhibited a structural diversity of *d* = 0.89 (*n *= 2,741), a value identical to that observed for Pezizomycotina SMs. Thus, SMs produced by *Aspergillus* and by Pezizomycotina fungi appear to be as structurally diverse as those of bacterial producers.

The similar structural diversity values between *Aspergillus* and the broader Pezizomycotina may suggest that *Aspergillus* SMs are as diverse as the rest of the sampled structural diversity observed in the subphylum. One explanation is that the similarity of values stems from sampling artifacts or biases. *Aspergillus* SMs account for 28% of Pezizomycotina SMs, so the observed similarity could simply be a sampling artifact. However, the diversity metric for all Pezizomycotina SMs in MIBiG, if one excludes *Aspergillus,* remains 0.89, suggesting that this explanation is unlikely. Nevertheless, these estimates are derived from manually curated BGC-SM pairs in the MIBiG database, which are likely to introduce detection biases. MIBiG is enriched for SMs that are bioactive, experimentally tractable, and pharmacologically relevant and may underrepresent rare, lineage-specific, or ecologically specialized SMs. This curation bias could compress observable diversity differences between lineages and potentially underestimate the true diversity within less-studied clades.

Another possibility is that biosynthetic space in Pezizomycotina is nearing saturation (i.e., new species contribute fewer novel scaffolds as more are sampled); this is supported by the finding that inclusion of *Aspergillus* SMs did not increase the structural diversity of Pezizomycotina. Furthermore, *Aspergillus* is an ancient and ecologically highly diverse genus that contains many prolific SM producers (23, 45). Evolutionary pressures such as balancing or diversifying selection may favor the maintenance or generation of multiple chemically distinct SMs across lineages, often because different compounds confer advantages in distinct ecological contexts and help maintain high SM diversity within *Aspergillus*.

## CONCLUSIONS AND FUTURE DIRECTIONS

We estimate that the known species of filamentous fungi in the Pezizomycotina subphylum may collectively produce at least 1.4 million SMs, which represents an immense reservoir of unexplored chemical diversity. Considering that we have only characterized ~30,000 fungal SMs to date, most SMs, even in well-studied genera such as *Aspergillus*, remain uncharacterized. These estimates depict the large opportunity for discovery of novel bioactive compounds that may have applications in medicine, agriculture, and industry.

While we focused our efforts to estimate SM diversity in Pezizomycotina, SMs are also biosynthesized by many other groups of fungi (20). For example, the red pigment pulcherrimin chelates iron and immobilizes it in the environment, inhibiting the growth of species such as *Botrytis cinerea*, and is produced from several different species of Saccharomycotina yeasts in the phylum Ascomycota (46, 47), whereas the well-known neuroactive compound psilocybin is produced by species of *Psilocybe* gilled mushrooms (48) and the lethal amatoxins are produced by the death cap mushroom *Amanita phalloides* (49), both of which are in the Basidiomycota phylum. SMs are also produced by fungi outside of the sub-kingdom Dikarya (Ascomycota and Basidiomycota). For example, certain Zoopagomycota fungi produce indole alkaloids that inhibit the growth of oomycete phytopathogens, whereas many Mucoromycota fungi produce carotenoid pigments, especially β-carotene, which protects fungal cells from free radicals, preventing oxidative stress damage, and are also commercially important colorants for food products and cosmetics (50–53). Therefore, the diversity of fungal SMs is likely even larger than our estimates.

Many fungal BGCs are transcriptionally silent under standard laboratory conditions, making discovery through expression-based approaches more challenging (54). As a result, genome mining has become central to exploring fungal chemodiversity. Tools such as antiSMASH, CASSIS (55), and DeepBGC (56) have expanded BGC detection by leveraging conserved domain architectures, promoter motif analysis, and machine learning, respectively. Integrative approaches that combine genomic predictions with chemical similarity data have further improved BGC-SM associations, enabling the prioritization of candidate clusters for novel scaffolds (57).

Despite the advances in bioinformatics and genome mining tools, many BGCs remain undetected due to enzymatic novelty or atypical genomic organization. For example, the isocyanide synthase gene cluster was identified through comparative genomics and experimental validation and only later incorporated into antiSMASH (58). Additionally, due to the set of strict rules antiSMASH employs, it also fails to detect many types of fungal ribosomally synthesized and post-translationally modified peptides, beyond a few well-characterized groups, as their precursor and tailoring enzyme can be dispersed or arranged atypically in the genome (59). Similarly, in *A. niger*, a silent azaphilone BGC was only characterized after promoter exchange enabled expression and metabolite identification (40). These cases highlight both the power and limitations of current genome mining frameworks. While bioinformatic tools have dramatically expanded our ability to explore fungal biosynthetic potential, a substantial portion of SM diversity likely remains hidden without complementary comparative, integrative, or experimental strategies.

In an era where high-throughput, systematic collection of genomic and metabolomic data is increasingly feasible, we are in the opportune position to dramatically expand the frontiers of fungal SM discovery. The increasing availability of integrative approaches that combine genomic sequencing and metabolomics will not only aid in characterizing novel SMs and the breadth of fungal chemodiversity but also link chemotype (SMs) with genotype (their BGCs), a major unresolved challenge (54). As saturation trends in BGC discovery likely differ by biosynthetic class (e.g., non-ribosomal peptide synthetases [NRPS], polyketide synthases [PKS], terpene) and taxonomic lineage, class- and clade-specific rarefaction analyses may provide more accurate estimates of undiscovered diversity and guide prioritization for targeted discovery efforts.

Our estimate is intended as a conservative lower bound based on currently available genomic and chemical data from a highly studied genus and existing literature. Because genome mining tools like antiSMASH define BGCs based on domain-level signatures, they can miss atypical clusters or combine functionally distinct pathways. We minimized overestimation by focusing on GCFs rather than individual BGCs and used a conservative SM-per-BGC ratio derived from curated BGC-SM pairs. Additionally, processes such as horizontal gene transfer and gene duplication (19, 60), which can significantly expand biosynthetic potential, were not included in our modeling and may further increase the true diversity. While the full scope of fungal SM diversity remains unknown, our framework provides a baseline for quantifying and comparing biosynthetic potential across fungal lineages. If our estimates are in the right ballpark, it appears that for every SM we currently know, there are ~50–100 additional SMs that are waiting for discovery. Thus, fungal SMs are a potential goldmine in our quest for new drugs, biotechnological advancements, and ecological insights into the fascinating biology of fungal organisms.

## Data Availability

All supplemental methods, genome accession numbers, and data files required to replicate these analyses have been deposited to the figshare repository (https://doi.org/10.6084/m9.figshare.29900843).
